# Outcomes With Venetoclax 50 mg, Hypomethylating Agents, and Voriconazole or Posaconazole in Acute Myeloid Leukemia

**DOI:** 10.1002/jha2.70049

**Published:** 2025-05-06

**Authors:** Kendall Diebold, Devan Parker, Sarah Worth, Manuel Espinoza‐Gutarra, Pankit Vachhani, Kimo Bachiashvili, Sravanti Rangaraju, Razan Mohty, Ravi Bhatia, Omer Jamy

**Affiliations:** ^1^ Division of Hematology and Oncology, Department of Medicine University of Alabama at Birmingham Birmingham Alabama USA

**Keywords:** acute myeloid leukemia (AML), posaconazole, Venetoclax 50 mg, voriconazole

## Abstract

**Background:**

Real‐world evidence for hypomethylating agent (HMA) + venetoclax 50 mg (VEN50) with voriconazole and posaconazole in acute myeloid leukemia (AML), is limited.

**Methods:**

We evaluated outcomes of patients with newly‐diagnosed AML treated with HMA + VEN50 with either posaconazole (*n* = 23) or voriconazole (*n* = 95).

**Results:**

We report that treatment with HMA + VEN50 with either azole elicits a response rate similar to that described in the VIALE‐A trial. Reducing the VEN dose to 50 mg with either strong CYP3A4 inhibitor did not compromise on the efficacy of the combination.

**Conclusion:**

HMA + VEN50 with either posaconazole or voriconazole yields comparable responses to higher doses of VEN reported previously.

**Clinical trial registration:**

The authors have confirmed clinical trial registration is not needed for this submission.

1

The combination of hypomethylating agents (HMA) and venetoclax (VEN) is the standard induction regimen for older or unfit patients with newly diagnosed acute myeloid leukemia (AML) [1]. The incidence of documented or suspected fungal infection in patients receiving HMA + VEN is approximately 3%–6%. However, given that myelosuppression, especially prolonged neutropenia, is a common adverse event of HMA + VEN, antifungal prophylaxis is often implemented with a mold‐active azole [2, 3]. Strong CYP3A4 inhibitors are commonly used for fungal prophylaxis in cases of prolonged neutropenia. Although posaconazole (compared to itraconazole or fluconazole) is the only agent that has demonstrated superior outcomes in AML, voriconazole is an acceptable alternative in cases of intolerability or high costs associated with posaconazole [2, 4, 5]. CYP3A4 is responsible for the metabolism of VEN. As posaconazole and voriconazole inhibit CYP3A4, they interact with the metabolism of VEN, leading to the need for dose‐adjustments for these combinations. A posaconazole drug‐drug interaction study showed that both the 50‐ and 100‐mg VEN doses administered with posaconazole were well tolerated and recommended a VEN dose reduction by at least 75% [6]. Such studies for voriconazole combination are not reported to our knowledge. In the VIALE‐A clinical trial, patients receiving strong CYP3A4 inhibitors received VEN 50 mg (VEN50). However, only 15% and 6% of the patients treated with HMA + VEN received posaconazole and voriconazole, respectively [1]. In contrast, the recommendation by the US Food and Drug Administration (FDA), as noted in the prescribing package insert of VEN, is to reduce the dose of VEN to 70 mg with posaconazole and 100 mg with voriconazole. Recently Kawedia et al. reported that VEN50 is an appropriate dose reduction when combined with posaconazole on the basis of comparable PK parameters observed with VEN without a strong CYP3A4 inhibitor. Their patient population was from a trial of VEN, cladribine, and LDAC for AML. Real‐world clinical data for HMA + VEN50 with voriconazole and posaconazole is very limited. Herein we share our clinical experience of HMA + VEN50 with both posaconazole and voriconazole in newly diagnosed AML.

We conducted a retrospective analysis to evaluate outcomes of patients with newly diagnosed AML treated with HMA + VEN50 with either posaconazole or voriconazole at the University of Alabama at Birmingham (UAB) between September 2021 and April 2024.

Variables captured included age at diagnosis, gender, race and ethnicity, and risk stratification per ELN 2022 and 2024 classification. Outcomes of interest included CR rate, CR with incomplete hematological recovery (CRi) rate, morphological leukemia‐free state (MLFS), overall response rate (ORR) (CR/CRi/MLFS), and overall survival (OS). Measurable residual disease (MRD) was assessed using flow cytometry validated to a sensitivity level of 0.1%. Starting treatment with HMA consisted of azacitidine 75 mg/m^2^ for 7 days or decitabine 20 mg/m^2^ for 5 days per cycle. VEN was administered at 50 mg, without ramp‐up, for 28 days for the first cycle, followed by 21–28 days or less for subsequent cycles, depending on disease response.

We identified 95 patients that received HMA + VEN50 with concomitant voriconazole for newly diagnosed AML (Table 1). The median age was 72 years (range 47–88 years). Majority of the patients were white (66.3%) and male (51.5%). Most patients had de novo AML (56.5%). By ELN 2022 classification, the breakdown of favorable, intermediate, and adverse‐risk disease was 13.6%, 12.6%, and 73.8%, respectively. By ELN 2024 classification, the breakdown was 32.6%, 30.5%, and 36.9%, respectively. All but one patient received decitabine.

**TABLE 1:** Characteristics and outcomes.

	Voriconazole (*n* = 95)	Posaconazole (*n* = 23)
**Median age (years)**	72	72
Range (years)	47–88	57–59
**Gender (%)**		
Male	51.5	47.9
Female	48.5	52.1
**Caucasian (%)**	66.3	95.6
***De novo *AML (%)**	56.5	60.8
**ELN 2022 (%)**		
Favorable	13.6	4.3
Intermediate	12.6	17.3
Adverse	73.8	78.4
**ELN 2024 (%)**		
Favorable	32.6	47.8
Intermediate	30.5	30.4
Adverse	36.9	21.8
**First cycle response rate (CR/CRi/MLFS) (%)**	38.9	52.2
CR (%)	20	15
**Best overall response rate (%)**	62.1	60.8
CR (%)	41.1	39.1
**Allogenic stem cell transplant rate (%)**	6	9
**30‐day mortality rate (%)**	12.6	8.6

Abbreviation: MLFS, morphological leukemia‐free state.

The ORR (CR/CRi/MLFS) after the first cycle of induction was 38.9%, with a CR rate of 20%. The best ORR obtained was 62.1% (median two cycles) with a CR rate of 41.1%. For comparison, the best overall response in the VIALE‐A trial was 66.4% with a CR rate of 36.7%. Eighty patients had MRD evaluation, and the rate of MRD‐negative response in our population was 22.1% after a median of one cycle (range 1–5). Only 6% of the patients proceeded with allogeneic stem cell transplantation. The 30‐day mortality rate was 12.6%. Out of these 12 patients, 4 patients died within the first 5 days of starting cycle 1 and would not have met enrollment criteria per VIALE‐A eligibility. In VIALE‐A, the 30‐day mortality was 7% with azacitidine‐VEN and 6% with azacitidine alone.

The median overall survival (mOS) for the entire group was 10 m. When analyzed by ELN 2024, the mOS for favorable, intermediate, and adverse‐risk disease was 13, 14, and 4 m, respectively.

Twenty‐three patients received HMA + VEN50 with concomitant posaconazole for newly diagnosed AML (Table 1). The median age was 72 years (range 57–79 years). Majority of the patients were white (95.6%) and female (52.1%). Most patients had de novo AML (60.8%). By ELN 2022 classification, the breakdown of favorable, intermediate, and adverse‐risk disease was 4.3%, 17.3%, and 78.4%, respectively. By ELN 2024 classification, the breakdown was 47.8%, 30.4%, and 21.8%, respectively. All patients received decitabine.

The ORR (CR/CRi/MLFS) after the first cycle of induction was 52.2% with a CR rate of 15%. The best ORR obtained was 60.8% (median two cycles) with a CR rate of 39.13%. Nineteen patients had MRD evaluation, and the rate of MRD‐negative response was 17.4% after a median of one cycle (range 1–4). Overall, 9% of the patients proceeded with allogeneic stem cell transplantation. The 30‐day mortality rate was 8.6% and the mOS was 11 m for the entire cohort.

We report that patients with AML treated with HMA + VEN50 with either posaconazole or voriconazole have a response rate similar to that described in the VIALE‐A trial. Reducing the dose to 50 mg with either strong CYP3A4 inhibitor did not compromise on the efficacy of the combination. Although much consideration is given to reducing the dose of VEN with posaconazole, our report shows that a lower dose of VEN is feasible to administer with voriconazole as well when given for 28 days.

Strong CYP3A4 inhibitors may increase levels of VEN in the blood, leading to excessive toxicity. Current recommendations by the US FDA include a dose of 70 mg of VEN when combined with posaconazole and 100 mg when combined with voriconazole. Most clinical trials being designed today, including those with triplet combinations, are following these guidelines. Our data suggests that a VEN dose of 50 mg may be sufficient with either of these azoles.

A concern of lower dose VEN is decreased efficacy. Limited data exists on clinical outcomes with VEN50, especially with voriconazole. A VEN dose of 100 mg is more commonly used with voriconazole. Our sample size of 95 patients receiving voriconazole and VEN50 demonstrated similar ORR to VIALE‐A, alleviating some of those concerns. Although our OS (10 m with voriconazole and 11 m with posaconazole) is lower than that reported in VIALE‐A, it is in line with the results of several real‐world analysis where the median OS has ranged from 7.9 to 13.6 m [3]. Most of those studies administered VEN similar to VIALE‐A. Furthermore, as real‐world studies offer insights into a much broader and less‐controlled setting, the outcomes may often be lower than clinical trials.

All patients in our analysis received VEN50 for 28 days during the first cycle. With evolving data, a shorter duration of VEN, ranging from 7 to 21 days per cycle, is being considered. At our institution, we are investigating a shorter duration of VEN50 per cycle. It is worth noting that the time to reach a steady state for posaconazole and voriconazole is approximately 7–10 and 3–5 days, respectively [7, 8]. Outcomes of VEN50 with posaconazole or voriconazole, when given for a shorter duration, will need to be further investigated in the context of the time taken to reach a steady state for these azoles. It is not clear if the efficacy of VEN50 will be compromised due to sub‐therapeutic levels of the drug in shorter courses and whether the timing of initiating the azole in the cycle will have to be reevaluated. Additionally, our findings are limited to HMA + VEN combination. The use of triplet regimens, although not currently a standard, may further alter the PK levels of VEN either directly or indirectly through the alteration of azole levels, as previously shown with at least ivosidenib [9].

In conclusion, HMA + VEN50 (28 days) with either posaconazole or voriconazole yields comparable responses to higher doses of VEN reported previously. In addition to larger validation of these results, future research should focus on the pharmacokinetic interactions of strong CYP3A4 inhibitors with shorter durations of VEN.

## Author Contributions

Kendall Diebold and Omer Jamy contributed toward the conception of the presented study and data collection and analysis. All authors contributed toward drafting, revising, and approving the manuscript.

## Ethics Statement

Institutional IRB approval was obtained.

## Conflicts of Interest

The authors declare no conflicts of interest.

## Data Availability

The data that support the findings of this study are available on request from the corresponding author.
